# Supplementary material for: Cytokine gene polymorphism and parasite susceptibility in free-living rodents: Importance of non-coding variants
Source: PLoS One. 2023 Jan 24;18(1):e0258009. doi: 10.1371/journal.pone.0258009 (PMC9873194; doi:10.1371/journal.pone.0258009)
Supplement: S1 Table — The primers included degenerated sites (marked in bold). (PDF) [file pone.0258009.s001.pdf]

**S1**. PCR conditions and sequences of the primers used in the current study. The primers included degenerated sites (marked in bold).

| | primer sequence 5’ → 3’ | Tm (°C) | exten-sion (sec) | cycles | source |
|---|---|---|---|---|---|
| | Detection of pathogens in blood | | | | |
| *Bartonella sp.* | F: AATGCAAAAAGAACAGTAAACA<br>R: TTACTTATGATCC**K**GG**Y**TTTA | 54 | 120 | 45 | Norman et al. 1995 |
| *Babiesia microti* | PCR1<br>F: GAATGATCCTTCCGCAGGTTCACCTAC<br>R: AACCTGGTTGATCCTGCCAGTAGTCAT | 58 | 120 | 35 | Persing et al. 1992 |
| | PCR2 (nested)<br>F: CTTAGTATAAGCTTTTATACAGC<br>R: ATAGGTCAGAAACTTGAATGATACA | 55 | 120 | 35 | Persing et al. 1992 |
| | Amplification of cytokines | | | | |
| TNF | F: ACCATGAGCACAGAAAGCAT<br>R: CTTCTCCAGCTGGAAGACT | 67 | 60 | 35 | Turner et al. 2012 |
| LTα | F: CTCTACCTCTTGAGGGTGCT<br>R: GTGTG**W**GTGGACAGCTGGTC | 66 | 60 | 30 | Kloch and Biedrzycka 2020 |
| IFNβ1 | F: CAGGTGGATCCTCCAAGCTGC<br>R: CT**Y**ATTCCACCCAGTGCTGG | 70 | 60 | 35 | current work |
