# Supplementary material for: Cytokine gene polymorphism and parasite susceptibility in free-living rodents: Importance of non-coding variants
Source: PLoS One. 2023 Jan 24;18(1):e0258009. doi: 10.1371/journal.pone.0258009 (PMC9873194; doi:10.1371/journal.pone.0258009)
Supplement: S2 Table — Number of respective exons and introns in mouse is given in parentheses. In TNF and LTα after slash we provide polymorphism summarises for exonic parts only. (PDF) [file pone.0258009.s002.pdf]

S2. Characteristic of the studied amplicons and summary of polymorphisms within the studied genes. Number of respective exons and introns in mouse is given in parentheses. In *TNF* and *LTα* after slash we provide polymorphism summarises for exonic parts only.

	TNF	***LTα***	***IFNβ1***
genotyped fragment (bp)	858	817	286
number of exons	3 (4)	3 (4*)	1 (1)
number of introns	2 (3)	2 (3)	0 (0)
number of segregating sites (S)	18 / 2	17 / 10	8
number of haplotypes	39 / 3	43 / 15	8
nucleotide diversity (π)	0.0045 / 0.0008	0.0046 / 0.0027	0.012
haplotype diversity (H_{div})	0.937 / 0.303	0.921 / 0.628	0.564
average no of nucleotide differences (k)	3.846 / 0.310	3.249 / 1.426	3.47

*) Three exons (2-4) are transcribed in mouse. First exon contains only UTR.
