# Supplementary material for: Cytokine gene polymorphism and parasite susceptibility in free-living rodents: Importance of non-coding variants
Source: PLoS One. 2023 Jan 24;18(1):e0258009. doi: 10.1371/journal.pone.0258009 (PMC9873194; doi:10.1371/journal.pone.0258009)
Supplement: S3 Table — We removed missing calls, variants with MAF<0.05, not in Hardy-Weinberg equilibrium (threshold p<0.001), and in linkage disequilibrium (r> 0.7). (PDF) [file pone.0258009.s003.pdf]

S3. Number of voles with given genotypes after filtering. We removed missing calls, variants with MAF<0.05, not in Hardy-Weinberg equilibrium (threshold p<0.001), and in linkage disequilibrium (r> 0.7).

locus	SNP	genotypes		
TNF	TNF 1431	**A|A**	**A|G**	**G|G**
		5	11	51
LTα	LTα 322	**C|C**	**C|T**	**T|T**
		1	17	102
	LTα 347	**A|A**	**G|A**	**G|G**
		106	13	1
	LTα 371	**A|A**	**G|A**	**G|G**
		72	43	5
	LTα 389	**C|C**	**C|T**	**T|T**
		2	41	77
	LTα 348	**A|C**	**C|C**	
		14	106	
	LTα 488	**C|C**	**C|T**	**T|T**
		1	15	104
	LTα 525	**G|G**	**T|G**	**T|T**
		38	51	31
IFNβ1	IFNβ1 105	**C|C**	**T|C**	**T|T**
		37	34	14
	IFNβ1 127	**A|A**	**A|G**	**G|G**
		21	33	31
