# Supplementary material for: Cytokine gene polymorphism and parasite susceptibility in free-living rodents: Importance of non-coding variants
Source: PLoS One. 2023 Jan 24;18(1):e0258009. doi: 10.1371/journal.pone.0258009 (PMC9873194; doi:10.1371/journal.pone.0258009)
Supplement: S4 Table — The number of infected animals differs between studied genes because not all individuals were genotyped in three loci. non-inf–number of non-infected hosts, inf–number of infected hosts, %–percentage of host infected. (PDF) [file pone.0258009.s004.pdf]

S4. Prevalence of infections among bank voles. The number of infected animals differs between studied genes because not all individuals were genotyped in three loci. non-inf – number of non-infected hosts, inf – number of infected hosts, % – percentage of host infected.

	TNF			***LTα***			***IFNβ1***		
**parasite / pathogen**	**non-inf**	**inf**	**%**	**non-inf**	**inf**	**%**	**non-inf**	**inf**	**%**
Nematodes (N) and cestodes (C)									
Catenotaenia hentonenni (C)	64	3	4.48	106	8	7.01	75	10	11.76
Aspiculuris tianjensis (N**)**	32	35	52.24	67	40	37.38	64	21	24.71
Capillaria sp. (N)	64	3	4.48	111	3	2.63	0	0	0.00
Heligmosomum mixtum (N)	52	15	22.39	74	40	35.09	50	35	41.18
Heligmosomoides glareoli (N)	59	8	11.94	92	15	14.02	66	19	22.35
Masophorus muris (N)	64	3	4.47	110	6	5.45	82	3	3.53
Intestinal protozoa									
Cryptosporidium sp.	14	33	70.21	40	51	56.05	36	45	55.56
Blood parasites									
Babesia microti	51	12	19.05	87	22	20.18	66	17	20.48
Bartonella sp.	44	17	27.87	75	32	29.90	61	22	26.51
