# Supplementary material for: Cytokine gene polymorphism and parasite susceptibility in free-living rodents: Importance of non-coding variants
Source: PLoS One. 2023 Jan 24;18(1):e0258009. doi: 10.1371/journal.pone.0258009 (PMC9873194; doi:10.1371/journal.pone.0258009)
Supplement: S5 Table — Terms with p<0.1 (marked in bold) were included in GLM models testing for the effect of genetic variance (S7 Table). The models were run separately on three datasets, each including voles genotyped at a given locus (not all animals were genotyped in all loci). (PDF) [file pone.0258009.s005.pdf]

S5. Summary of effect of non-genetic terms on parasite load. Terms with p<0.1 (marked in bold) were included in GLM models testing for the effect of genetic variance (S6). The models were run separately on three datasets, each including voles genotyped at a given locus (not all animals were genotyped in all loci).

gene	parasite	site	year	host body mass	host sex
**Presence/absence**					
TNF n=67	*H. mixtum*	**<0.001**	**0.078**	**0.072**	0.599
	A. tianjensis	**0.017**	0.910	0.340	0.515
	Cryptosporidium sp.	**0.091**	-	0.394	**0.008**
	Babesia microti	0.742	0.275	0.466	**0.080**
	Bartonella sp.	0.233	**0.016**	0.573	0.930
LTα n=114	*H. mixtum*	**<0.001**	**0.003**	**0.018**	0.776
	A. tianjensis	**<0.001**	0.532	**0.080**	0.509
	Cryptosporidium sp.	**0.001**	-	0.139	0.162
	Babesia microti	0.896	0.325	0.831	**0.019**
	Bartonella sp.	0.447	**0.049**	0.435	0.773
IFNβ1 n=85	*H. glareoli*	**<0.001**	-	**0.089**	**0.000**
	H. mixtum	**<0.001**	-	**0.009**	0.771
	A. tianjensis	**0.001**	-	0.107	0.379
	Cryptosporidium sp.	**0.001**	-	**0.066**	0.244
	Babesia microti	0.713	-	0.576	**0.088**
	Bartonella sp.	0.852	-	0.515	0.766
**Abundance**					
TNF n=67	*H. mixtum*	**0.079**	0.483	**0.042**	0.133
	A. tianjensis	0.999	0.128	0.776	0.912
LTα n=114	*H. mixtum*	**<0.001**	0.621	**0.094**	0.322
	A. tianjensis	0.179	**0.002**	0.360	0.657
IFNβ1 n=85	*H. glareoli*	**0.000**	-	0.975	**0.001**
	H. mixtum	**<0.001**	-	**0.039**	0.175
	A. tianjensis	**<0.001**	-	**0.002**	0.152
