# Supplementary material for: Cytokine gene polymorphism and parasite susceptibility in free-living rodents: Importance of non-coding variants
Source: PLoS One. 2023 Jan 24;18(1):e0258009. doi: 10.1371/journal.pone.0258009 (PMC9873194; doi:10.1371/journal.pone.0258009)
Supplement: S6 Table — As response variables we used only pathogens that infected 20–80% of hosts (S4 Table). β is parameter estimate for each contrast, R2 is partial coefficient of determination (effect size), χ2 and p-values are based on LR type III test. To control for multiple comparisons when testing for the effect of several genetic variants, we used conservative Bonferroni correction; for 10 genetic terms (SNPs) tested, the critical p-level corresponding to α = 0.05 was 0.005. Exact p-values of genetic terms significant after correction are given in bold. (PDF) [file pone.0258009.s006.pdf]

**S6.** Effect on cytokine genetic variants on parasite load. As response variables we used only pathogens that infected 20-80% of hosts (S4). β is parameter estimate for each contrast, $R^2$ is partial coefficient of determination (effect size), $\chi^2$ and p-values are based on LR type III test. To control for multiple comparisons when testing for the effect of several genetic variants, we used conservative Bonferroni correction; for 10 genetic terms (SNPs) tested, the critical p-level corresponding to α=0.05 was 0.005. Exact p-values of genetic terms significant after correction are given in bold.

| **Presence / absence** | | | | | | | |
|---|---|---|---|---|---|---|---|
| **locus** | **response** | **variables** | **β** | **$R^2$** | **$\chi^2$** | **df** | **p** |
| *TNF* n=67 | *H. mixtum* | TNF 1431 | A\|G: -16.757<br>G\|G: -17.574 | 0.060 | 2.520 | 2 | 0.284 |
| | | year | -1.6233 | 0.105 | 4.597 | 1 | 0.032 |
| | | site | Tałty: 35.008<br>Urwitałt: 36.299 | 0.292 | 25.182 | 2 | <0.001 |
| | | host body mass | 0.127 | 0.030 | 2.419 | 1 | 0.120 |
| | *A. tianjinensis* | TNF 1431 | A\|G: -0.765<br>G\|G: -0.801 | 0.011 | 0.592 | 2 | 0.744 |
| | | site | Tałty: 1.229<br>Urwitałt: -0.778 | 0.139 | 8.908 | 2 | 0.012 |
| | *Cryptosporidium sp.* | TNF 1431 | A\|G: 17.3017<br>G\|G: -16.9151 | 0.054 | 3.435 | 2 | 0.180 |
| | | site | Tałty: -1.0312<br>Urwitałt: -2.2199 | 0.064 | 4.245 | 2 | 0.120 |
| | | host sex | -2.0615 | 0.156 | 8.336 | 1 | 0.004 |
| | *Babesia microti* | TNF 1431 | A\|G: 14.7205<br>G\|G: 15.8719 | 0.025 | 2.530 | 2 | 0.282 |
| | | host sex | -0.8616 | 0.025 | 1.710 | 1 | 0.191 |
| | *Bartonella sp.* | TNF 1431 | A\|G: 0.829<br>G\|G: 0.056 | 0.021 | 0.954 | 2 | 0.621 |
| | | year | 1.357 | 0.079 | 4.001 | 1 | 0.046 |
| *LTα* n=114 | *H. mixtum* | LTα 322 | T\|T: 0.311 | 0.000 | 0.053 | 1 | 0.819 |
| | | LTα 347 | G\|A: -17.186 | 0.009 | 1.371 | 1 | 0.242 |
| | | LTα 371 | G\|A: 17.768 | 0.000 | 0.000 | 1 | 1.000 |
| | | LTα 389 | T\|T: 16.7332 | 0.000 | 0.000 | 1 | 1.000 |
| | | LTα 411 | C\|C: 0.820 | 0.013 | 0.896 | 1 | 0.344 |
| | | LTα 488 | T\|T: 0.5349 | 0.000 | 0.494 | 1 | 0.482 |
| | | LTα 525 | T\|G: -0.444<br>T\|T: 0.840 | 0.050 | 4.893 | 2 | 0.087 |
| | | site | Tałty: 18.416<br>Urwitałt: 20.372 | 0.340 | 54.968 | 2 | <0.001 |
| | | year | -1.418 | 0.050 | 5.299 | 1 | 0.021 |
| | | host body mass | 0.1581 | 0.057 | 6.584 | 1 | 0.010 |

| | | | | | | |
|---|---|---|---|---|---|---|
| *A. tianjinensis* | LTα 322 | T\|T:-18.449 | 0.090 | 7.735 | 1 | 0.005 |
| | LTα 347 | G\|A: -17.365 | 0.027 | 2.918 | 1 | 0.088 |
| | LTα 371 | G\|A: -2.765 | 0.031 | 2.287 | 1 | 0.130 |
| | LTα 389 | T\|T: 0.466 | 0.001 | 0.086 | 1 | 0.770 |
| | LTα 411 | C\|C:0.689 | 0.005 | 0.513 | 1 | 0.474 |
| | LTα 488 | T\|T: -0.208 | 0.002 | 0.059 | 1 | 0.808 |
| | LTα 525 | T\|G: -1.3774<br>T\|T: -1.990 | 0.051 | 4.580 | 2 | 0.101 |
| | site | Tałty: 1.159<br>Urwitałt: -2.668 | 0.271 | 26.167 | 2 | <0.001 |
| | host body mass | 0.1316 | 0.018 | 2.906 | 1 | 0.088 |
| *Cryptosporidium sp.* | LTα 322 | T\|T: -15.837 | 0.021 | 2.622 | 1 | 0.105 |
| | LTα 347 | G\|A: -17.085 | 0.040 | 3.812 | 1 | 0.051 |
| | LTα 371 | G\|A: 0.825 | 0.003 | 0.207 | 1 | 0.649 |
| | LTα 389 | T\|T: 1.154 | 0.008 | 0.479 | 1 | 0.489 |
| | LTα 411 | C\|C: 0.730 | 0.002 | 0.645 | 1 | 0.422 |
| | LTα 488 | T\|T: 0.945 | 0.015 | 1.434 | 1 | 0.231 |
| | LTα 525 | T\|G: 0.338<br>T\|T: -1.021 | 0.050 | 4.128 | 2 | 0.127 |
| | site | Tałty: -0.7692<br>Urwitałt: -1.883 | 0.101 | 8.410 | 2 | 0.015 |
| *Babesia microti* | LTα 322 | T\|T: 14.867 | 0.009 | 1.042 | 1 | 0.307 |
| | LTα 347 | G\|A: 14.920 | 0.010 | 0.921 | 1 | 0.337 |
| | LTα 371 | G\|A: 1.207 | 0.004 | 0.344 | 1 | 0.558 |
| | LTα 389 | T\|T: 0.816 | 0.003 | 0.190 | 1 | 0.663 |
| | LTα 411 | C\|C: -0.994 | 0.028 | 1.878 | 1 | 0.171 |
| | LTα 488 | T\|T: 0.066 | 0.000 | 0.008 | 1 | 0.930 |
| | LTα 525 | T\|G: 1.744<br>T\|T: 1.334 | 0.043 | 4.561 | 2 | 0.102 |
| | host sex | -1.261 | 0.050 | 5.355 | 1 | 0.021 |
| *Bartonella sp.* | LTα 322 | T\|T: -1.041 | 0.003 | 0.498 | 1 | 0.481 |
| | LTα 347 | G\|A: -1.143 | 0.003 | 0.377 | 1 | 0.539 |
| | LTα 371 | G\|A: -0.649 | 0.001 | 0.108 | 1 | 0.742 |
| | LTα 389 | T\|T: 0.752 | 0.001 | 0.172 | 1 | 0.678 |
| | LTα 411 | C\|C: -1.129 | 0.026 | 2.249 | 1 | 0.134 |
| | LTα 488 | T\|T: -0.479 | 0.007 | 0.462 | 1 | 0.497 |
| | LTα 525 | T\|G: -0.294<br>T\|T: -1.065 | 0.026 | 2.254 | 2 | 0.324 |
| | year | 1.166 | 0.028 | 2.901 | 1 | 0.089 |

| | | | | | | | |
|---|---|---|---|---|---|---|---|
| | *H. glareoli* | IFNβ1 105 | T\|C: 0.3866<br>T\|T: 22.911 | 0.267 | 30.025 | 2 | **3.21x10$^{-7}$** |
| | | IFNβ1 127 | A\|G:19.420<br>G\|G: 19.744 | 0.077 | 7.636 | 2 | 0.022 |
| | | site | Urwitałt: -22.016 | 0.492 | 91.094 | 1 | <0.001 |
| | | host body mass | -0.270 | 0.084 | 7.149 | 1 | 0.007 |
| | | host sex | -2.693 | 0.191 | 17.498 | 1 | <0.001 |
| | *H. mixtum* | IFNβ1 105 | T\|C: -17.485<br>T\|T: -17.079 | -0.001 | 0.095 | 2 | 0.953 |
| | | IFNβ1 127 | A\|G: -18.472<br>G\|G: -34.962 | 0.021 | 2.168 | 2 | 0.338 |
| | | site | Urwitałt: 39.999 | 0.690 | 129.850 | 1 | <0.001 |
| | | host body mass | 0.199 | 0.127 | 7.023 | 1 | 0.008 |
| *IFNβ1*<br>n=85 | *A. tianjinensis* | IFNβ1 105 | T\|C: 0.0375<br>T\|T: 0.1433 | 0.000 | 0.007 | 2 | 0.996 |
| | | IFNβ1 127 | A\|G: 1.374<br>G\|G: 1.756 | 0.022 | 1.593 | 2 | 0.451 |
| | | site | Urwitałt: -2.259 | 0.179 | 14.645 | 1 | <0.001 |
| | *Cryptosporidium sp.* | IFNβ1 105 | T\|C: 0.1547<br>T\|T: 0.0220 | 0.027 | 2.331 | 2 | 0.312 |
| | | IFNβ1 127 | A\|G: 1.296<br>G\|G: 1.916 | 0.016 | 1.238 | 2 | 0.538 |
| | | site | Urwitałt: -2.675 | 0.105 | 7.588 | 1 | 0.006 |
| | | host body mass | 0.132 | 0.050 | 3.923 | 1 | 0.048 |
| | *Babesia microti* | IFNβ1 105 | T\|C: 0.927<br>T\|T: -0.076 | 0.015 | 1.076 | 2 | 0.584 |
| | | IFNβ1 127 | A\|G: -0.764<br>G\|G: -0.272 | 0.006 | 0.565 | 2 | 0.754 |
| | | host sex | -1.295 | 0.056 | 4.233 | 1 | 0.040 |
| | *Bartonella sp.* | IFNβ1 105 | T\|C: 17.068<br>T\|T: 15.363 | 0.067 | 6.751 | 2 | 0.034 |
| | | IFNβ1 127 | A\|G: -1.152<br>G\|G: 15.473 | 0.038 | 4.293 | 2 | 0.117 |

| Abundance | | | | | | | |
|---|---|---|---|---|---|---|---|
| **locus** | **response** | **variables** | **β** | **$R^2$** | **$\chi^2$** | **df** | **p** |
| *TNF*<br>n=67 | *H. mixtum* | TNF 1431 | A\|G: -2.381<br>G\|G: -1.287 | 0.043 | 1.296 | 2 | 0.523 |
| | | site | Tałty: 18.267<br>Urwitałt: 18.415 | 0.075 | 5.951 | 2 | 0.051 |
| | | host body mass | 0.056 | 0.013 | 0.500 | 1 | 0.480 |

| | | | | | | | |
|---|---|---|---|---|---|---|---|
| | *A. tianjinensis* | TNF 1431 | A\|G: 0.505<br>G\|G: 1.935 | 0.031 | 3.199 | 2 | 0.202 |
| *LTα*<br>n=114 | *H. mixtum* | LTα 322 | T\|T: 0.256 | 0.001 | 0.051 | 1 | 0.822 |
| | | LTα 347 | G\|A: -16.843 | -0.002 | 2.039 | 1 | 0.153 |
| | | LTα 371 | G\|A: 17.086 | 0.000 | 0.000 | 1 | 1.000 |
| | | LTα 389 | T\|T: 15.888 | 0.000 | 0.000 | 1 | 1.000 |
| | | LTα 411 | C\|C:0.312 | 0.001 | 0.334 | 1 | 0.563 |
| | | LTα 488 | T\|T: 0.274 | 0.009 | 0.287 | 1 | 0.592 |
| | | **LTα 525** | T\|G: -0.967<br>T\|T: 0.384 | **0.046** | **12.480** | **2** | **0.00195** |
| | | site | Tałty: 18.583<br>Urwitałt: 19.865 | 0.124 | 30.689 | 2 | <0.001 |
| | | host body mass | 0.051 | 0.023 | 2.779 | 1 | 0.095 |
| | *A. tianjinensis* | LTα 322 | T\|T: -1.881 | 0.012 | 2.136 | 1 | 0.144 |
| | | LTα 347 | G\|A: -1.499 | 0.021 | 1.103 | 1 | 0.294 |
| | | LTα 371 | G\|A: -1.502 | -0.005 | 0.319 | 1 | 0.572 |
| | | LTα 389 | T\|T: -0.774 | -0.001 | 0.082 | 1 | 0.775 |
| | | LTα 411 | C\|C: 0.410 | 0.012 | 0.228 | 1 | 0.633 |
| | | LTα 488 | T\|T: 0.126 | 0.002 | 0.017 | 1 | 0.896 |
| | | LTα 525 | T\|G: 0.297<br>T\|T: -0.404 | 0.006 | 0.913 | 2 | 0.633 |
| | | year | 1.411 | 0.054 | 5.720 | 1 | 0.017 |
| *IFNβ1*<br>n=85 | *H. glareoli* | **IFNβ1 105** | T\|C: -1.309<br>T\|T: 17.571 | **0.136** | **12.986** | **2** | **0.00151** |
| | | **IFNβ1 127** | A\|G: 18.301<br>G\|G: 16.835 | **0.172** | **12.546** | **2** | **0.00189** |
| | | site | Urwitałt: -19.599 | 0.180 | 42.039 | 1 | <0.001 |
| | | host sex | -1.353 | 0.127 | 10.558 | 1 | 0.001 |
| | *H. mixtum* | IFNβ1 105 | T\|C: 0.0689<br>T\|T: -0.889 | 0.010 | 2.254 | 2 | 0.324 |
| | | IFNβ1 127 | A\|G: -0.692<br>G\|G: 0.0554 | 0.057 | 2.517 | 2 | 0.284 |
| | | site | Urwitałt: 20.401 | 0.315 | 103.287 | 1 | <0.001 |
| | | mass | 0.061 | 0.067 | 8.208 | 1 | 0.005 |
| | *A. tianjinensis* | IFNβ1 105 | T\|C: 0.452<br>T\|T: 2.813 | -0.010 | 1.815 | 2 | 0.404 |
| | | IFNβ1 127 | A\|G: 0.545<br>G\|G: 3.133 | 0.038 | 2.683 | 2 | 0.261 |
| | | site | Urwitałt: -5.018 | 0.110 | 22.697 | 1 | <0.001 |
| | | host body mass | 0.204 | 0.048 | 8.858 | 1 | 0.003 |
