# Supplementary material for: Cytokine gene polymorphism and parasite susceptibility in free-living rodents: Importance of non-coding variants
Source: PLoS One. 2023 Jan 24;18(1):e0258009. doi: 10.1371/journal.pone.0258009 (PMC9873194; doi:10.1371/journal.pone.0258009)
Supplement: S7 Table — Only exonic parts of the studied genes are analysed. Codons were numbered starting from the first genotyped nucleotide, not from the first transcribed nucleotide. Names of the corresponding SNPs, as used in the current paper, are given in brackets. Codons that comprised SNPs significantly associated with the parasite load are given in bold. (PDF) [file pone.0258009.s007.pdf]

S7. Codons under selection. Only exonic parts of the studied genes are analysed. Codons were numbered starting from the first genotyped nucleotide, not from the first transcribed nucleotide. Names of the corresponding SNPs, as used in the current paper, are given in brackets. Codons that comprised SNPs significantly associated with the parasite load are given in bold.

	positive			negative	
gene	FUBAR	FEL	MEME	FUBAR	FEL
TNF	–	–	–	74<br>91	–
LTα	42<br>(=SNP371)<br>46	–	–	48<br>59	48<br>59
IFNβ1	**39 (=SNP 127)**	**–**	**–**	**31 (=SNP 105)**	**31 (=SNP 105)**
