# Supplementary material for: Cytokine gene polymorphism and parasite susceptibility in free-living rodents: Importance of non-coding variants
Source: PLoS One. 2023 Jan 24;18(1):e0258009. doi: 10.1371/journal.pone.0258009 (PMC9873194; doi:10.1371/journal.pone.0258009)
Supplement: S8 Table — β is parameter estimate for each contrast, R2 is partial coefficient of determination (effect size), χ2 and p-values are based on LR type III test. To control for multiple comparisons when testing for the effect of several genetic variants, we used conservative Bonferroni correction; for 10 genetic terms (SNPs) tested, the critical p-level corresponding to α = 0.05 was 0.005. Exact p-values of genetic terms significant after correction are given in bold. (PDF) [file pone.0258009.s008.pdf]

S8. Effect on cytokine genetic variants on parasite load with all non-genetic terms fitted. β is parameter estimate for each contrast, R^2 is partial coefficient of determination (effect size), χ^2 and p-values are based on LR type III test. To control for multiple comparisons when testing for the effect of several genetic variants, we used conservative Bonferroni correction; for 10 genetic terms (SNPs) tested, the critical p-level corresponding to α=0.05 was 0.005. Exact p-values of genetic terms significant after correction are given in bold.

Presence / absence							
**locus**	**response**	**variables**	**β**	**R^2**	**χ^2**	**df**	**p**
TNF n=67	*H. mixtum*	TNF1431	A|G: -17.123<br>G|G: -17.826	0.065	2.541	2	0.281
		year	-1.549	0.098	3.812	1	0.051
		site	Tałty: 35.036<br>Urwitałt: 36.334	0.298	24.139	1	0.000
		host sex	-0.442	0.010	0.373	1	0.542
		host body mass	0.110	0.022	1.579	1	0.209
	A. tianjinensis	TNF 1431	A|G: -0.573<br>G|G: -0.671	0.007	0.361	2	0.835
		year	0.082	0.000	0.010	1	0.921
		site	Tałty: 1.157<br>Urwitałt: -0.896	0.136	8.129	1	0.017
		host sex	0.299	0.005	0.209	1	0.648
		host body mass	0.075	0.014	0.793	1	0.373
	Cryptosporidium sp.	TNF 1431	A|G: 17.297<br>G|G: -16.857	0.052	3.329	2	0.189
		site	Tałty: -1.026<br>Urwitałt: -2.027	0.052	3.293	2	0.193
		host sex	-2.366	0.170	8.838	1	0.003
		host body mass	-0.085	0.017	0.736	1	0.391
	Babesia microti	TNF 1431	A|G: 14.446<br>G|G: 15.795	0.031	2.454	2	0.293
		year	1.143	0.027	1.392	1	0.238
		site	Tałty: -0.436<br>Urwitałt: -0.989	0.009	0.763	2	0.683
		host sex	-1.276	0.055	2.768	1	0.096
		host body mass	-0.079	0.036	0.596	1	0.440
	Bartonella sp.	TNF 1431	A|G: 1.187<br>G|G: 0.595	0.027	0.654	2	0.721
		year	2.311	0.137	5.221	1	0.022
		site	Tałty: -1.144<br>Urwitałt: -1.759	0.067	2.334	2	0.311
		host sex	0.245	0.009	0.088	1	0.767
		host body mass	0.078	0.011	0.452	1	0.502

LTα n=114	*H. mixtum*	LTα 322	T|T: 0.201	0.000	0.021	1	0.884
		LTα 347	G|A:-17.191	0.009	1.340	1	0.247
		LTα 371	G|A:17.671	0.000	0.000	1	1.000
		LTα 389	T|T: 16.549	0.000	0.000	1	1.000
		LTα 411	C|C: 0.913	0.016	1.113	1	0.291
		LTα 488	T|T: 0.712	0.002	0.781	1	0.377
		LTα 525	T|G: -0.476<br>T|T: 0.894	0.058	5.312	2	0.070
		year	-1.335	0.039	4.351	1	0.037
		site	Tałty: 18.395<br>Urwitałt: 20.399	0.344	53.775	2	0.000
		host sex	-0.512	0.012	0.987	1	0.320
		host body mass	0.153	0.055	5.890	1	0.015
	A. tianjinensis	LTα 322	T|T: -18.393	0.080	6.771	1	0.009
		LTα 347	G|A: -17.272	0.023	2.569	1	0.109
		LTα 371	G|A: -2.778	0.032	2.152	1	0.142
		LTα 389	T|T: 0.428	0.000	0.068	1	0.795
		LTα 411	C|C: 0.683	0.005	0.465	1	0.495
		LTα 488	T|T: -0.195	0.002	0.047	1	0.829
		LTα 525	T|G: -1.372<br>T|T: -1.995	0.052	4.288	2	0.117
		year	-0.315	0.007	0.082	1	0.775
		site	Tałty: 1.246<br>Urwitałt: -2.627	0.272	4.314	2	0.000
		host sex	0.110	0.001	0.032	1	0.858
		host body mass	0.136	0.018	2.609	1	0.106
	Cryptosporidium sp.	LTα 322	T|T: -17.416	0.038	3.856	1	0.050
		LTα 347	G|A: -18.404	0.049	4.482	1	0.034
		LTα 371	G|A: 0.247	0.000	0.018	1	0.894
		LTα 389	T|T: 0.715	0.004	0.181	1	0.670
		LTα 411	C|C: 0.917	0.007	1.009	1	0.315
		LTα 488	T|T: 1.157	0.025	2.077	1	0.149
		LTα 525	T|G: -1.096<br>T|T: 0.423	0.060	4.813	2	0.090
		site	Tałty: -0.816<br>Urwitałt: -1.992	0.097	8.729	2	0.013
		host sex	-0.954	0.035	2.990	1	0.084
		host body mass	-0.131	0.032	3.223	1	0.073

	Babesia microti	LTα 322	T|T: 16.255	0.015	1.277	1	0.258
		LTα 347	G|A: 16.431	0.015	1.173	1	0.279
		LTα 371	G|A: 1.417	0.006	0.414	1	0.520
		LTα 389	T|T: 0.985	0.005	0.241	1	0.623
		LTα 411	C|C: -1.017	0.024	1.669	1	0.196
		LTα 488	T|T: 0.120	0.000	0.022	1	0.882
		LTα 525	T|G: 1.649<br>T|T: 1.934	0.054	4.488	2	0.106
		year	0.513	0.008	0.370	1	0.543
		site	Tałty: 0.493<br>Urwitałt: -0.062	0.005	0.488	2	0.783
		host sex	-1.321	0.039	4.797	1	0.029
		host body mass	0.024	0.005	0.130	1	0.718
	Bartonella sp.	LTα 322	T|T: -1.094	0.006	0.481	1	0.488
		LTα 347	G|A: -1.479	0.007	0.550	1	0.458
		LTα 371	G|A: -0.631	0.001	0.092	1	0.761
		LTα 389	T|T: 0.774	0.002	0.164	1	0.686
		LTα 411	C|C: -0.982	0.021	1.514	1	0.219
		LTα 488	T|T: -0.528	0.006	0.446	1	0.504
		LTα 525	T|G: -0.265<br>T|T: -0.884	0.016	1.148	2	0.563
		year	1.490	0.041	3.587	1	0.058
		site	Tałty: -0.840<br>Urwitałt: -0.556	0.018	1.214	2	0.545
		host sex	0.204	0.004	0.148	1	0.700
		host body mass	0.047	0.008	0.471	1	0.492
IFNβ1<br>n=85	*H. glareoli*	IFNβ1 105	T|C: 0.386<br>T|T: 22.911	0.267	30.025	2	**$3.2x10^{-7}$**
		IFNβ1 127	A|G: 19.421<br>G|G: 19.745	0.077	7.636	2	0.022
		site	22.016	0.492	91.094	1	<0.001
		host sex	-2.694	0.084	7.149	1	0.007
		host body mass	-0.270	0.191	17.498	1	0.000
	H. mixtum	IFNβ1 105	T|C: -17.629<br>T|T: -17.184	-0.001	0.117	2	0.943
		IFNβ1 127	A|G: -18.519<br>G|G: -35.101	0.022	2.374	2	0.305
		site	40.028	0.690	133.055	1	<0.001
		host sex	0.257	0.124	7.194	1	0.007
		host body mass	0.196	0.000	0.172	1	0.678

	A. tianjinensis	IFNβ1 105	T|C: -0.005<br>T|T: 0.057	0.000	0.002	2	0.999
		IFNβ1 127	A|G: 1.481<br>G|G: 1.919	0.028	1.426	2	0.490
		site	-2.783	0.239	14.517	1	0.000
		host sex	0.700	0.078	3.443	1	0.064
		host body mass	0.164	0.028	0.938	1	0.333
	Cryptosporidium sp.	IFNβ1 105	T|C: -0.266<br>T|T: 1.358	0.022	1.785	2	0.410
		IFNβ1 127	A|G: 0.832<br>G|G: 0.052	0.013	0.931	2	0.628
		site	-1.518	0.110	8.016	1	0.005
		host sex	-0.496	0.055	4.414	1	0.036
		host body mass	-0.130	0.010	0.746	1	0.388
	Babesia microti	IFNβ1 105	T|C: 0.905<br>T|T: -0.196	0.016	1.089	2	0.580
		IFNβ1 127	A|G: 0.903<br>G|G: -0.354	0.009	0.717	2	0.699
		site	0.206	0.003	0.109	1	0.742
		host sex	-1.179	0.005	0.340	1	0.560
		host body mass	0.038	0.043	3.110	1	0.078
	Bartonella sp.	IFNβ1 105	T|C: 17.111<br>T|T: 15.266	0.070	6.762	2	0.034
		IFNβ1 127	A|G: -1.276<br>G|G: 15.461	0.045	4.559	2	0.102
		site	0.0617	0.001	0.012	1	0.913
		host sex	-0.047	0.005	0.596	1	0.440
		host body mass	0.049	0.000	0.007	1	0.932

Abundance							
**locus**	**response**	**variables**	**β**	**R^2**	**χ^2**	**df**	**p**
TNF<br>n=67	*H. mixtum*	TNF 1431	A|G: -1.146<br>G|G: -0.299	0.009	0.344	2	0.842
		year	0.459	0.005	0.389	1	0.533
		site	Tałty: 17.855<br>Urwitałt: 17.969	0.028	4.504	2	0.105
		sex	1.0168	0.018	1.349	1	0.246
		mass	0.135	0.027	2.188	1	0.139

	A. tianjinensis	TNF 1431	A|G: 0.650<br>G|G: 2.122	0.077	3.425	2	0.180
		year	1.204	0.071	3.323	1	0.068
		site	Tałty: -0.441<br>Urwitałt: -0.173	0.024	0.364	2	0.834
		sex	0.202	0.005	0.112	1	0.738
		mass	0.033	-0.003	0.157	1	0.692
LTα<br>n=114	*H. mixtum*	LTα 322	T|T: 0.356	0.003	0.094	1	0.759
		LTα 347	G|A:-16.726	-0.004	1.755	1	0.185
		LTα 371	G|A:17.059	0.000	0.000	1	1.000
		LTα 389	T|T:15.846	0.000	0.000	1	1.000
		LTα 411	C|C:0.272	-0.001	0.231	1	0.631
		LTα 488	T|T: 0.229	0.007	0.177	1	0.674
		LTα 525	T|G: -0.964<br>T|T: 0.361	0.033	11.055	2	**0.00397**
		year	-0.171	0.010	0.160	1	0.689
		site	Tałty:18.606<br>Urwitałt:19.880	0.108	27.650	2	0.000
		sex	0.154	0.006	0.229	1	0.632
		mass	0.052	0.029	2.684	1	0.101
	A. tianjinensis	LTα 322	T|T: -1.942	0.006	2.820	1	0.093
		LTα 347	G|A:-1.765	0.009	1.879	1	0.170
		LTα 371	G|A:-1.558	0.002	0.436	1	0.509
		LTα 389	T|T:-0.831	0.002	0.121	1	0.728
		LTα 411	C|C:0.480	0.007	0.355	1	0.552
		LTα 488	T|T: 0.224	0.002	0.061	1	0.804
		LTα 525	T|G: 0.517<br>T|T: 0.344	0.012	0.716	2	0.699
		year	2.301	0.029	9.947	1	0.002
		site	Tałty:-1.144<br>Urwitałt:-1.530	0.010	4.117	2	0.128
		sex	0.104	-0.002	0.035	1	0.851
		mass	0.085	0.000	1.144	1	0.285
IFNβ1<br>n=85	*H. glareoli*	IFNβ1 105	T|C: -1.334<br>T|T: 17.563	0.174	12.924	2	**0.00156**
		IFNβ1 127	A|G: 18.338<br>G|G: 16.826	0.216	12.582	2	**0.00185**
		site	-19.671	0.238	39.713	1	0.000
		sex	-1.265	0.052	0.255	1	0.613

	mass	0.0263	-0.020	7.129	1	0.008
H. mixtum	IFNβ1 105	T|C: -0.038<br>T|T: -1.029	0.016	2.592	2	0.274
	IFNβ1 127	A|G: -0.849<br>G|G: -0.187	0.055	2.610	2	0.271
	site	20.461	0.321	103.514	1	< 0.000
	sex	0.065	0.095	8.847	1	0.003
	mass	0.360	0.045	2.569	1	0.109
A. tianjinensis	IFNβ1 105	T|C: 0.099<br>T|T: 2.631	0.000	2.419	2	0.298
	IFNβ1 127	A|G: 1.023<br>G|G: 3.203	0.018	1.918	2	0.383
	site	-5.831	0.097	28.406	1	0.000
	sex	1.560	0.026	12.641	1	0.000
	mass	0.359	-0.016	3.123	1	0.077
