# Supplementary material for: Cytokine gene polymorphism and parasite susceptibility in free-living rodents: Importance of non-coding variants
Source: PLoS One. 2023 Jan 24;18(1):e0258009. doi: 10.1371/journal.pone.0258009 (PMC9873194; doi:10.1371/journal.pone.0258009)

S1. Parasite load in individuals genotyped in a) *TNF*, b) *LTα*, and c) *IFNβ1*. Dark bars represent infected animals, light – non-infected.

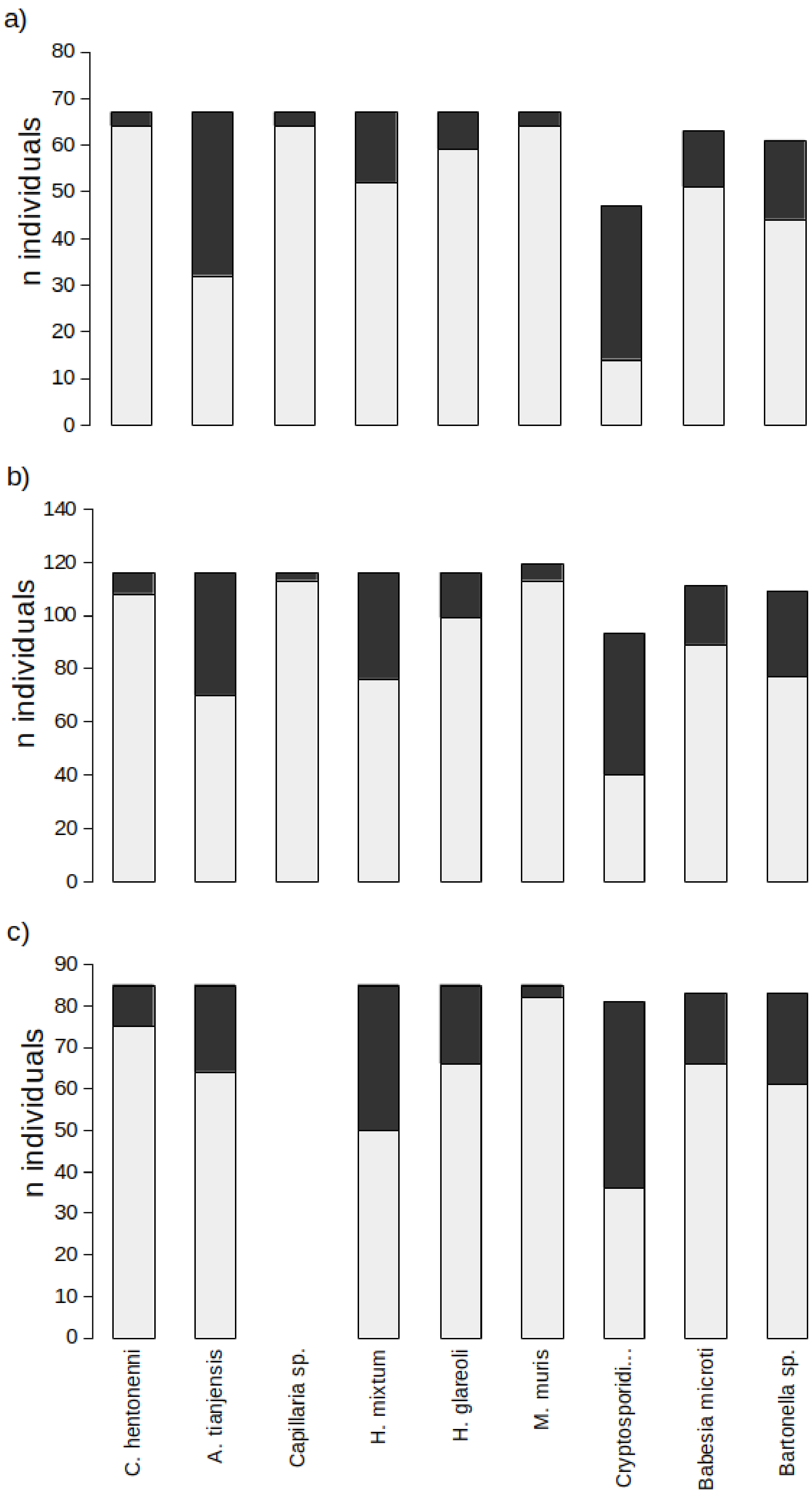

Supplement: S1 Fig — Parasite load by parasite species in individuals genotyped in a) TNF, b) LTα, and c) IFNβ1. Dark bars represent infected animals, light–non-infected. (PDF) [file pone.0258009.s009.pdf]
