# Supplementary material for: Cytokine gene polymorphism and parasite susceptibility in free-living rodents: Importance of non-coding variants
Source: PLoS One. 2023 Jan 24;18(1):e0258009. doi: 10.1371/journal.pone.0258009 (PMC9873194; doi:10.1371/journal.pone.0258009)
Supplement: Attachment — Submitted filename: 2response_rev.docx [file pone.0258009.s011.docx]

**Reviewer #1**:

*Changes in text following Reviewer’s #1 suggestions are marked in blue.*

*We refer to line numbers in “track changes” version of the revised manuscript.*

I appreciate that authors have carefully addressed my comments and significantly improved the quality of the manuscript.

Thank you. We are glad that we managed to satisfy the Reviewer remarks.

I now have one major comment concerning positive selection analysis that I unfortunately missed previously. Positive selection analysis cannot be performed on exon-intron sequences and authors need to redo the analysis only on CDS regions and re-interpret results where necessary.

Thank you for pointing out this important mistake. We calculated the tests again using only exonic fragments, changing relevant parts in Methods and Results (lines 290-297). (There were no changes in IFNb1, as it consisted of exonic part only).

1) L49, Parasites may affect the genetic variation of their hosts through three main mechanisms.
There also some other „main“ mechanisms, such positive or purifying selection. Better opening sentence for BS is needed.

We agree with the remark, although summarizing such a complex idea in a single sentence is challenging. Nonetheless, we attempted to better introduce BS (lines 48-50).

2) L127, All three sites are on public ground managed by the Polish State Forests 128 and no specific permission to access the land was required. This sentence ca be omitted.

This sentence was added upon request of the Editor: „In your Methods section, please provide additional information regarding the permits you obtained for the work. Please ensure you have included the full name of the authority that approved the field site access and, if no permits were required, a brief statement explaining why.”

3) L171, A fraction of SNPs (5 of 36 in TNF, and 6 of 18 in LTα) could 172 not be phased by FreeBayes. To avoid confusion, better to use „reconstructed“ over „phased“. Also, better to write, a fraction of alleles…

By using the term „a fraction of SNPs” we wanted to precisely depict the situation. In the vcf file resulting from our pipeline most of the SNPs were phased (i.e. assigned to given DNA strand) based on their positions on sequenced Illumina fragments (reads). If two SNPs are located in the same read, the FreeBayes assumes that they originated from the same DNA strand. This method does not work for SNPs that are separated from other SNPs by more than a read length and thus never occurr in a read with any other SNP. Here we had to use PHASE algorithm and assigned them computationally to given DNA strands.

We clarified the sentence, so now it reads “a fraction of SNPs (5 of 36 in *TNF*, and 6 of 18 in *LTα*) could not be phased by FreeBayes; these were computationally assigned to DNA strands using PHASE (Stephens et al. 2001). Reconstructed alleles were converted to fasta format ..” (lines 188-190).

4) Table 1 can be better placed in SI as it shows only supportive information.
The table was initially in the SI but was placed in the main text upon request of the Reviewer 3: „Materials and methods, section 2.1: It would be important to add here the sample size of voles in the two years at each site into the main text (rather than a supplementary table).” We can move it back to the SI if both Reviewers (#1 and #3) agree.

5) L233, We overcome this drawback by using two additional tests (MEME and FUBAR) to test for episodic selection. FUBAR detect only pervasive selection. Please, correct the statement.

We agree with this remark. After consideration, we decided to remove two final sentences of this paragraph, as the principles of all the tests are described above. We kept the information that FEL assumes constant selection pressure across phylogeny.

5) L238-239 should be moved to the methods.

This passage was added upon request of the Reviewer #1 in the first round of reviews: “ *P6, 3.1. Cytokine polymorphisms and susceptibility to infections. I would add some general summary of polymorphism revealed, ideally with a table in SI with values such as number of sequences, number of unique nucleotide alleles, number of variable sites, number of substitutions to see how much are cytokines variable.” Nontheless, in the current review, we moved it to the end of section 2.2, lines 196-199.

6) L346-349. This is redundant sentence as mechanism of NFSD is already well-explained in the introduction.

This was added upon request of Reviewer #1: „P8, L7 Please, clarify what is meant by repeated rounds of positive selection interspersed with purifying selection.”. However, as we the explanation of NFSD was also added to the introduction, we removed this redundant fragment.

7) L357, Given the absence of revealed recombination, those results must be interpreted with caution. Without direct functional testing using mouse models, the revealed association between parasite load and particular SNPs must be interpreted with caution as it can be caused by some other adjacent variation. Please, incorporate this point in the discussion.

We agree. We added this point to the discussion, lines 373-376.

7) L642,
It seems that authors analysed pattern of positive selection across whole exon-intron sequences. This is misleading since dN/dS based selection method are intended only for protein-coding data. Introns are under different mode of evolution. I ask authors to perform selection analyses only on protein-coding data, i.e. CDS region and change the interpretation of data where necessary. Also, please, explicitly mention in the methods that selection analysis was performed only on exonic sequences
Thank you for pointing out this mistake. We have now recalculated site-selection tests using exonic sequences only in LTa and TNF (INFb1 did not contain introns). We changed relevant parts of Methods (l. 226) and Results (section 3.2), including Fig 2.

8) Figure 2
Why do authors not provide location of sites under selection also for TNF?

Initially in Fig 2 we included only the genes where we find significant associations of given SNPs with parasite load. We agree that this may be confusing so we added TNF to the Figure.

**Reviewer #3**:

*Changes in text following Reviewer’s #2 suggestions are marked in green.*

*We refer to line numbers in “track changes” version of the revised manuscript.*

I reviewed the earlier version of the manuscript and I am pleased to see that the majority of coments and suggestions from the reviewers have been addressed. I would like to highlight, however, that the reviewing process would have been easier if the authors had added line numbers corresponding to each of their edits for each of their responses to the editors and reviewers comments.

Thank you for appreciation. In this review, we add line numbers corresponding to the edits.

However, I am still slightly skeptical about the associations since population structure can lead to false positives. Ideally, population structure would be accounted for in some way, or a set of control genes would also be included to check whether the test statistic (lambda) follows the null distribution or whether it is inflated. I am aware however that the authors do not have this data, and I am also aware that there are a lot of candidate gene studies out there that do not account for population structure, and I do believe that multiple testing correction in the revision has reduced the chance of false positives. As a result, I would be happy if the authors added some text to the discussion to indicate the caveats of this approach and the follow up studies needed to confirm any associations they see.

We are really thankful for understanding that some factors are difficult (or impossible) to control in the field data. We did our best to make our results as robust as possible. We added a paragraph discussing caveats of our study design in a final part of the section 4.1.

In terms of the non-genetic sources of variation to include in the model: I would usually advocate to keep all terms in the model as a more conservative approach. However, I understand that overfitting is a problem particularly when the sample sizes are low. I would trust the authors assertion that the models were similar if they added the alternative models with all fixed effects fitted (non-genetic sources and SNPs) to the supplement. I also think it would have been an easier approach to test – for each parasite and each parasite model (absence or parasite load) – the fixed effects rather than checking for each parasite model/gene subset as they could be significant in one and not the other just due to small changes in sample size.

We added models with all genetic terms in Table S7. Please note, that for IFN there is no factor “year”, as in this gene we only genotyped samples from 2005. Similarly, there is no “year” for *Cryptosporidium* which was analysed only in samples from 2005.

I do feel that the results are strengthened since applying the Bonferonni correction. I think ideally the number of tests would be - the number of parasites tested (5?) x number of parasite models (2) x number of genes (as SNPs within a gene might not be independent?) or SNPs. I would be interested to see what the other reviewers think of the criteria of 10 tests because I understand that my suggestion would be very very conservative. I would also like to note that given the current criteria the association between LTa 322 and A. tianjinensis is technically not significant (p=0.0054) and reference to this association should be removed.

Thank you for rising this point. Prior the publication, we discussed between us what should we consider *multiple comparisons.* For instance, we tested for linkage between SNPs within a locus as described in the first paragraph of the section 2.3. so technically they should be independent, yet statistically the more explanatory variables, the higher probability that any of them is significant by chance. Thus, we used the criterion of 10, as this was the number of explanatory variables whose effect we were interested in (the non-genetic terms were included only to control for their contribution to the observed variance in parasite load).

Probably it has to be clarified that due to a fact that prevalence was <20% in some combinations of loci and parasites, in total we run 23 models as shown in the table below. (This information was added in line 220). If we consider all combination of parasite x type of test x SNP as multiple comparisons, this gives in total 74 “comparisons” resulting in p-value 0.05 /74 = 0.00068. This super-conservative criterion still shows significant association between IFN*β* 105 and prevalence with *H. glareoli.*

Table. Combinations of parasites / genes tested in the GLM models. In total we run 23 tests. When each SNP is considered, this gives 74 comparisons.

|  | Presence / absence  16 models | | | Abundance  7 models | | |
| --- | --- | --- | --- | --- | --- | --- |
| parasite / pathogen | TNF | *LTα* | *IFNβ1* | TNF | *LTα* | *IFNβ1* |
| *Aspiculuris tianjensis* | 1 SNP | 7 SNP | 2 SNP | 1 SNP | 7 SNP | 2 SNP |
| *Heligmosomum mixtum* | 1 SNP | 7 SNP | 2 SNP | 1 SNP | 7 SNP | 2 SNP |
| *Heligmosomoides glareoli* | - | - | 2 SNP | - | - | 2 SNP |
| *Cryptosporidium sp.* | 1 SNP | 7 SNP | 2 SNP | - | - | - |
| *Babesia microti* | 1 SNP | 7 SNP | 2 SNP | - | - | - |
| *Bartonella sp.* | 1 SNP | 7 SNP | 2 SNP | - | - | - |

However, we doubt if models of abundance and presence/absence should be considered as multiple comparisons, as biologically each of them involves different hypothesis. Resistance against parasites involve different immunological mechanisms (i.e. preventing a pathogen from colonizing the hosts) than dealing with the number of pathogens once the infection happened. If we agree with this assumption, the threshold p-val for presence/absence tests is 0.05 / 52 (10 SNPs tested in 5 parasites + 2 SNPs tested for H. mixtum) = 0.00096 and for abundance this value is 0.05 / 22 (10 SNPs for two parasites plus 2 SNPs tested for H. mixtum) = 0.0023. Again, with those thresholds our results remain valid.

Finally, we think that corrections for multiple comparisons can be applied only to explanatory variables, as we are interested in minimizing risk of false positives. Models with different parasites species as response variables do not fulfil this criterion, as finding an association between genetic marker and susceptibility to disease A is independent from susceptibility to disease B - at least statistically, if we don’t assume any underlying biological mechanisms that may link these two diseases. Thus, we simply corrected for the number of loci tested. Such an approach is recommended in human GWAS studies, for instance in PLINK manual.

We agree that the association between LTa 322 and A. tianjinensis is weak and should not be reported so we adjusted Results and Discussion.

I would add the data availability statement into the manuscript

Initially, it was included in the PloS submission forms but it seems it did not appear in the manuscript. We added the statement to the main text. lines 451-455

You mention that you exclude variants (genotypes?) present in fewer than 5 animals but then I am unsure why LTa 322 is included since it has no CC genotype (or had 1 animal which was removed)?

We removed this one individual with the genotype CC from the analysis so that we still can test for the effect of LTa322.

I feel that the introduction is much improved by the addition of information about the study system. I do feel as though some hypotheses are a bit vague – as well as the statement that the authors focused on these genes as “little is known”. I did expect some more references to human GWAS which may have found SNPs associated with these genes and parasite/pathogen infections, although perhaps there are none. Here it would be better to specifically indicate the parasite species studied rather than e.g. ‘blood microparasites’.

We added some text to this paragraph to make it more specific. Although we did not intend to cite many human GWAS studies, as they usually have much power and different design compared to wild systems, we included a reference to a review by Khan and Qidwai 2011 (line 123). We rephrased the sentence in line 97 (“little is known”), and we reformulated the hypotheses.

Page 2, line 108 – parasite load of what species?

We rephrased the hypothesis and we made it more specific (lines 123-125 and 129-134).

The statistical methods are a lot clearer now, thank you.

We are happy that we succeeded to make the text clearer.

I appreciate the re-writing of the conclusion when I queried how important the non-coding variants are – I wonder also if this part of the title could be removed as you did find more coding variants.

We agree to remove “non-coding variants” from the title after acceptance from the Associated Editor.

I appreciate that the authors brought in the table of parasite species (Table 2) into the main manuscript in response to my query that I wanted the parasite species tested mentioned specifically in the text. However, I do feel like there is a lot of information in this table for the main manuscript. I would really prefer if the authors just listed the species studied directly in the text (referring to the type of parasite they are too) and put this table back in the Supp. I suggest this because it is much easier for readers to work out which species are looked at than having to look up the table. I also found the table difficult to interpret because I was unclear if parasite species were included if it was 20-80% infections across all gene subsets or only if in any – and if the latter – did you only run models where prevalence was > 20% for a given parasite/ gene combination (i.e. was H. glareoli only run with IFN variants and not the other two genes?). Therefore, I feel like it would be helpful to add the parasite species tested directly to the text as it was not immediately clear to me. If the authors elected to keep the table in the main manuscript you could remove the numbers infected and non-infected (as you could work this out from Table 1 from the % prevalence) to make it simpler. Either way I would highlight the species you tested in bold to help the reader.
We put Table 2 back in the Suppl Mat (now S4). We run only tests where prevalence was >20% for given gene/parasite combination, we clarified that in the text and we summarized the models tested (lines 220-226).

I would add the parasite species tested in the abstract. I imagine most people would be looking specifically for genetic variants associated with a given parasite (or closely related parasite) so this would help their search.

Added.

Page 3, line 74 – “non-coding variance” = non-coding variants

Corrected

Add how species abundance (not just prevalence) was measured to the methods.

Worms of each species were counted. The counts were used as abundance. We added this information in the line 135.

Add season/month of sample collection to methods.

Added. In both years it was September (line 138).

Methods - are quasi-binomial and quasi-poisson not two different types of model?

Yes, they are. The sentence may be confusing so we clarified “in abundance models we used quasi-Poisson errors and in presence models quasi-binomial errors” (line 235)

Results – I would add null results for each parasite/gene combination.

We added a suitable paragraph to the first part of the section 3.1. (lines 266-273)

Table 3 – I am glad the authors have put the full model results in the main manuscript. However, the formatting is not as nice as the old table and I am unclear why there are lines around host body mass. I would recommend adding a reference to the supp table with the other (non-sig) results to the legend. There is a reference to padj which isn’t reported in the table.

The lines were errors in editing. We corrected the caption. In the reviewed version we removed results for LTAa 322 (marginally non-significant, p=0.0052) which prompted us to reformat the table.

I think it would be nice to add to the summary at the start of the discussion which genes and which parasite species tested were not significant.

Added (lines 304-309)

Page 10, line 291 – change individuals to worms

Corrected

Page 10, line 298 – SNP variance = SNP variants

corrected to „variants”

Page 10, line 305 – generation *of* T cells

corrected

Page 10, line 310 – but you also investigated the association between TNF and other non-nematode infections?

Yes, we clarified this (line 337)

Page 11, line 330 – strengthened

corrected
